# Inhibitory Effects of *Siegesbeckia orientalis* Extracts on Advanced Glycation End Product Formation and Key Enzymes Related to Metabolic Syndrome

**DOI:** 10.3390/molecules22101785

**Published:** 2017-10-21

**Authors:** Wei-Chin Hung, Xue-Hua Ling, Chi-Chang Chang, Hsia-Fen Hsu, Shih-Wei Wang, Yi-Chen Lee, Ci Luo, Yun-Tzu Lee, Jer-Yiing Houng

**Affiliations:** 1 Division of Cardiology, E-Da Hospital, Kaohsiung 82445, Taiwan; ed102600@edah.org.tw; 2 Graduate Institute of Biotechnology and Chemical Engineering, I-Shou University, Kaohsiung 84001, Taiwan; ed107312@gmail.com; 3 Department of Obstetrics & Gynecology, E-Da Hospital/I-Shou University, Kaohsiung 82445, Taiwan; ed101779@edah.org.tw; 4 Department of Nutrition, I-Shou University, Kaohsiung 82445, Taiwan; fen153848@gmail.com (H.-F.H.); r9620239@yahoo.com.tw (C.L.); lisa10143018@gmail.com (Y.-T.L.); 5 Division of Allergy, Immunology, and Rheumatology, Department of Internal Medicine, E-Da Hospital/I-Shou University, Kaohsiung 82445, Taiwan; shihwei8888@gmail.com; 6 Department of Nutrition Therapy, E-Da Hospital, Kaohsiung 82445, Taiwan; ed103549@edah.org.tw

**Keywords:** *Siegesbeckia orientalis* linne, antioxidation, advanced glycation end products, α-amylase, α-glucosidase, lipase, angiotensin I-converting enzyme

## Abstract

Metabolic syndrome typically includes Type 2 diabetes associated with hyperglycemia, central obesity, dyslipidemia and hypertension. It is highly related to oxidative stress, formation of advanced glycated end products (AGEs) and key enzymes, such as carbohydrate digesting enzymes like pancreatic α-amylase and intestinal α-glucosidase, pancreatic lipase and angiotensin I-converting enzyme (ACE). This study used an in vitro approach to assess the potential of four extracts of *Siegesbeckia orientalis* linne on key enzymes relevant to metabolic syndrome. In this research, *S. orientailis* was firstly extracted by ethanol. The ethanol extract (SE) was then partitioned sequentially with hexane, ethyl acetate and methanol, and these extracts were named SE-Hex, SE-EA and SE-MeOH, respectively. The experimental results showed that SE-EA had the highest total phenolic content (TPC, 76.9 ± 1.8 mg/g) and the total flavonoids content (TFC, 5.3 ± 0.3 mg/g). This extract exhibited the most significant antioxidant activities, including DPPH radical-scavenging capacity (IC_50_ = 161.8 ± 2.4 μg/mL), ABTS radical-scavenging capacity (IC_50_ = 13.9 ± 1.5 μg/mL) and reducing power. For anti-glycation activities, SE-EA showed the best results in the inhibition of AGEs, as well as inhibitory activities against α-glucosidase (IC_50_ = 362.3 ± 9.2 μg/mL) and α-amylase (IC_50_ = 119.0 ± 17.7 μg/mL). For anti-obesity activities, SE-EA indicated the highest suppression effect on pancreatic lipase (IC_50_ = 3.67 ± 0.52 mg/mL). Finally, for anti-hypertension activity, SE-EA also demonstrated the strongest inhibitory activity on ACE (IC_50_ = 626.6 ± 15.0 μg/mL). Close relationships were observed among the parameters of TPC, antioxidant activities, inhibitory activities on α-amylase, α-glucosidase, lipase and ACE (R > 0.9). Moderate correlations were found among the parameters of TFC, antioxidant activities, and suppression of dicarbonyl compounds formation (R = 0.5–0.9). Taken together these in vitro studies reveal the therapeutic potential of SE-EA extract in the prevention and treatment of metabolic disorders.

## 1. Introduction

Metabolic syndrome is a cluster of conditions, including high blood sugar and/or blood pressure, excess body fat, and abnormal cholesterol or triglyceride levels. When these metabolic disorders occur together, the risk of Type 2 diabetes, cardiovascular disease and stroke increase significantly. Metabolic syndrome is closely linked to obesity and inactivity [1]. Insulin resistance is also a main risk factor for metabolic syndrome [2]. Therefore, the control of the development of Type 2 diabetes is generally the primary effort in clinical treatment on metabolic syndrome. 

Oxidative stress, induced by an abundance of reactive oxygen species (ROS) or failure in the antioxidative system, is a main cause of many diseases, such as diabetes, cancer, rheumatoid arthritis, atherosclerosis and aging [3]. Oxidation stress has been reported to be involved in the formation of advanced glycated end products (AGEs) [4,5,6]. In a variety of cells, AGEs trigger oxidative stress and inflammatory reactions through the interaction with the receptor for advanced glycation products. Consequently, it contributes to the development and progression of various metabolic disorders, such as cardiovascular disease, chronic kidney disease, and insulin resistance [7,8,9,10,11]. Therefore, scavenging the unwanted free radicals to keep the balance of the free radicals is necessary for controlling metabolic syndrome.

Glycation is a non-enzymatic reaction between the aldehyde group of sugars and the amino group of proteins, lipids, and nucleic acids, where it initially progresses to form the reversible Schiff bases. Subsequently, Schiff bases rearrange to form the more stable, covalently-bound Amadori products. Over a period of time, these early glycation products undergo further reactions, such as oxidation, dehydration and cyclization, leading to cumulative chemical modifications of proteins and resulting in the irreversible formation of AGEs [12,13]. The formation of AGEs will progressively accelerate under hyperglycemic, oxidative stress, and inflammatory conditions. Therefore, AGEs formation links to the progression of aging and the complications of diabetes, such as diabetic cardiovascular disease, diabetic nephropathy, diabetic peripheral neuropathy, diabetic ocular disease, and atherosclerotic disease [14,15]. Thus, suppression of glycation cascade has become a promising therapeutic approach for the prevention or treatment of diabetic or other pathogenic complications [16].

Another effective therapeutic strategy for Type 2 diabetes management is the use of inhibitors of carbohydrate digesting enzymes. The inhibition of pancreatic α-amylase and intestinal α-glucosidase decreases intestinal glucose digestion and absorption from starch and sucrose hydrolysis, thereby controlling the postprandial blood glucose elevation [17,18]. 

Obesity is a dominant pathogenic contributor to diabetes, cardiovascular diseases, and the combination of risk factors for metabolic syndrome [19,20,21]. Reducing the absorption of energy dense fats by inhibiting enzymes involved in lipid metabolism has been an effective therapeutic approach for obesity treatment [22]. Lipase is a key enzyme involved in triglyceride digestion, which hydrolyzes triglycerides into absorbable glycerol and fatty acids. The suppression of dietary lipid absorption by inhibiting the activity of pancreatic lipase has been a promising strategy for treating obesity [18]. 

Hypertension and Type 2 diabetes are metabolic disorders of a great relevance [23,24]. Sustained hypertension can cause strokes, heart attacks, congestive heart failure, and chronic renal failure [25]. Angiotensin I-converting enzyme (ACE) plays a crucial role in regulation blood pressure. ACE cleaves angiotensin I to produce angiotensin II, which is known as a powerful vasoconstrictor in hypertension. ACE also stimulates bradykinin release, which has a vasodilator function [26]. Therefore, ACE inhibitors have been widely prescribed for patients with both diabetic and non-diabetic symptoms to prevent angiotensin II production during the treatment of hypertension related to metabolic syndrome [27].

*Siegesbeckia orientalis* linne is a traditional Chinese herbal medicine with reported therapeutic effects on quadriplegia, rheumatoid arthritis, bone pain, waist and knee weakness, high blood pressure, and traumatic bleeding. Previous literature has reported that *S. orientalis* ethanol extract had immunosuppressive activity on ovalbumin in mice [28]. The ethyl acetate extract of *S. orientalis* could inhibit the proliferation of human cervical cancer HeLa cells [29]. Its ethanol extract could suppress the growth and metastasis of endometrial cancer cells [30,31]. Additionally, *S. orientalis* extracts possess significant anti-inflammatory, anti-hyperuricemic and analgesic activities [32,33]. However, to our best knowledge, the effects of *S. orientalis* extracts on metabolic syndrome, such as Type 2 diabetes-linked hyperglycemia and related cardiovascular complications, have not yet been reported in the literature. Based on the above rationale, the objective of this study was to evaluate the potential of *S. orientalis* extracts in the prevention or management of metabolic syndrome complications with their in vitro bioactivities.

## 2. Results and Discussion

### 2.1. Total Polyphenol and Flavonioid Content of S. orientalis Extracts

Abundant literature has reported that the phenolic compounds and flavonoids in vegetables, fruits and herbal medicines can scavenge free radicals and display high correlations with the antioxidation, antiglycemic activity, and inhibitory activities on α-amylase and α-glucosidase [34,35,36]. Polyphenols and flavonoids possess a protective effect on microvascular complications, such as kidney disease, chronic wounds, and other symptoms caused by oxidative stress. These ingredients have been considered in managing hyperglycemia and hypertension related to Type 2 diabetes [37,38,39]. Recently, a large amount of data from clinical and epidemiological studies has elucidated that polyphenols and flavonoids have received considerable interest for their roles in the prevention or treatment of various chronic diseases [40,41,42,43]. 

Numerous reports state that phenolic compounds extracted from some plants can be applied as part of a healthy diet to prevent diabetes. These phenolic compounds sources include fruit peels of *Nepheliuml appaceum* [44], and leaves of *Psidium guajava* Linn [45], *Peltophorum pterocarpum* [46], *Syzygium aqueum* [47] and *Lithocarpus polystachyus* Rehd [48]. For *S. orientalis*, Nguyen et al. [33] reported that its anti-hyperuricemic and anti-inflammatory effects related to the content of phenolic components, identified as caffeic acid analogues and flavonones.

**Figure 1:**
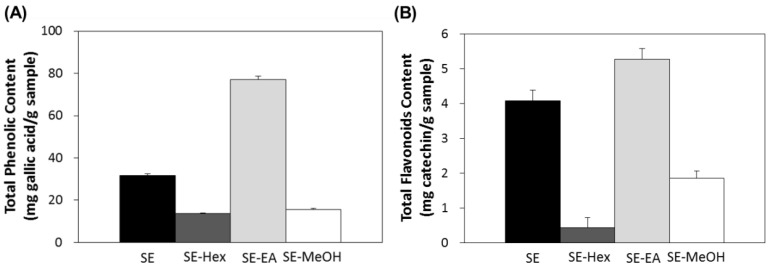
Total polyphenols content (**A**) and total flavonoids content (**B**) of *S. orientalis* extracts.

In this study, the aerial parts of *S. orientalis* were firstly extracted by ethanol and this ethanol extract (SE) was then partitioned sequentially with *n*-hexane, ethyl acetate and methanol to give the corresponding *n*-hexane extract (SE-Hex), ethyl acetate extract (SE-EA) and methanol extract (SE-MeOH), respectively. The total polyphenols content (TPC) and total flavonoids content (TFC) of these four extracts of *S. orientalis* were then measured (Figure 1). Among the extracts, the SE-EA contained the highest TPC (76.9 ± 1.8 mg/g) and TFC (5.3 ± 0.3 mg/g), followed by SE (31.7 ± 0.5 mg/g and 4.1 ± 0.3 mg/g), SE-MeOH (15.7 ± 0.4 mg/g and 1.9 ± 0.1 mg/g), and SE-Hex (13.7 ± 0.2 mg/g and 0.4 ± 0.3 mg/g). 

### 2.2. Antioxidant Effects

Uncontrolled production of oxygen free radicals has a variety of adverse pathological effects on many diseases including aging, diabetes, cardiovascular disease, cancer, and atherosclerosis. An excessive formation of free radicals may lead to the occurrence of oxidative stress. Increasing evidence reveals that increased oxidative stress is related to diabetes and cardiovascular disease [3,49]. Therefore, removing the excess free radicals is necessary to keep the balance of the free radicals.

**Figure 2:**
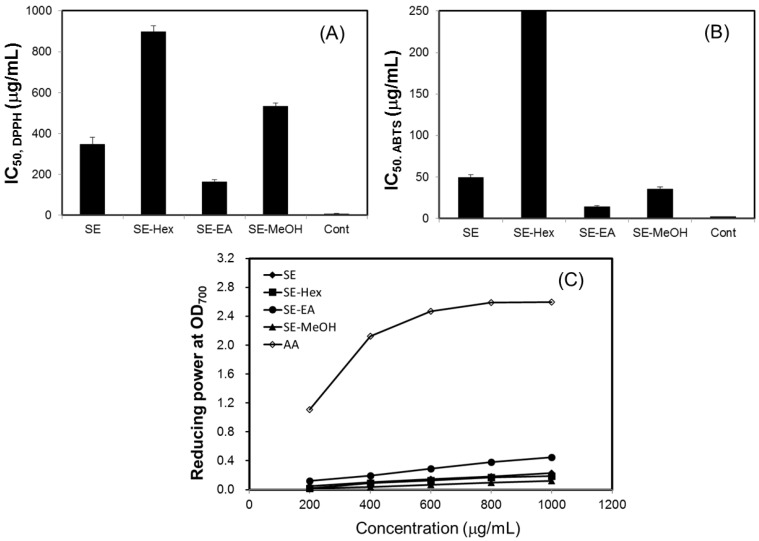
Antioxidant activity of *S. orientalis* extracts. (**A**) DPPH radical scavenging activity (expressed by IC_50_ values), catechin was used as the positive control; (**B**) ABTS radical cation scavenging activity (expressed by IC_50_ values), BHT was used as the positive control; (**C**) Reducing power, ascorbic acid (AA) was used as the positive control.

In this study, the antioxidant effects of *S. orientalis* extracts were evaluated by DPPH radical scavenging activity, ABTS radical cation scavenging capacity, and reducing power. For all extracts examined, within the concentration range tested, raising the concentration increased radicals scavenging activity. The IC_50_ values of DPPH and ABTS scavenging activities of different extracts were calculated (Figure 2A,B). The DPPH scavenging activities declined in the order of SE-EA (IC_50_ = 161.8 ± 2.4 μg/mL) > SE (IC_50_ = 346.6 ± 34.7 μg/mL) > SE-MeOH (IC_50_ = 533.4 ± 16.5 μg/mL) > SE-Hex (IC_50_ = 896.7 ± 9.6 μg/mL). For comparison, the IC_50_ values of DPPH scavenging activities of catechin, which was used as the positive control, was 6.8 ± 0.8 μg/mL. The ABTS scavenging activities followed the order of SE-EA (IC_50_ = 13.9 ± 1.5 μg/mL) > SE-MeOH (IC_50_ = 35.7 ± 2.6 μg/mL) > SE (IC_50_ = 49.1 ± 3.5 μg/mL), whereas SE-Hex showed only small ABTS scavenging activity. The IC_50_ of the positive control, BHT, was 2.3 ± 0.2 μg/mL. 

Figure 2C shows the reducing power of *S. orientalis* extracts using the potassium ferricyanide reduction method. The reducing power of SE-EA increased significantly with increasing concentration of samples. However, compared to the positive control, ascorbic acid, the reducing power of *S. orientalis* extracts was relatively low. Thus, *S. orientalis* extracts had high free radical scavenging activity, but low reducing power.

The antioxidant activities of many edible plant, fruits and vegetables increase as the TPC or TFC increases [50,51]. Table 1 shows the correlation coefficients (R) of various antioxidation activities of extracts with their respective TPC and TFC. Strong linear correlations existed between the reciprocal of IC_50_ value of DPPH and ABTS scavenging activity and reducing power with their respective TPC (R = 0.925–0.993), and mild correlations between these three bioactivities with TFC (R = 0.794–0.907). Additionally, the correlations between these three antioxidant activities were also good (R = 0.828–0.953).

### 2.3. Inhibitory Effects on AGEs Formation

**Figure 3:**
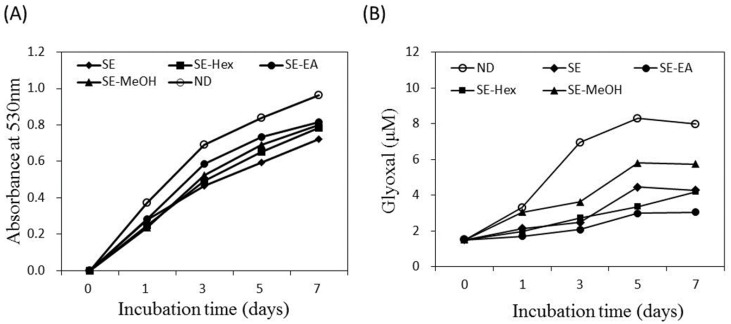
Inhibitory effect of *S. orientalis* extracts on the formation of AGEs. (**A**) Amadori products formation analyzed by the reduction of NBT; (**B**) Dicarbonyl compounds production analyzed by Girard-T assay. The concentration of each extract was 1 mg/mL.

Endogenous AGE formation contributes to the progression of pathogenesis associated with metabolic syndrome complications and aging [14,15]. The formation of Amadori products occurs at the early stage of non-enzymatic glycation. In the present study, the amount of Amadori products was analyzed by the reduction reaction with NBT and the colored product was detected at 530 nm. Figure 3A shows that the glycated-BSA caused an increase of optical density at 530 nm along the incubation time. The formation of Amadori products was inhibited by *S. orientalis* extracts. The inhibitory effects followed the order of SE > SE-Hex > SE-MeOH > SE-EA. The suppression rate of these extracts at the 7th day were 24.9%, 18.8%, 17.2% and 15.6%, respectively.

Dicarbonyl compounds can induce the cross-linking of proteins and produce around 45–50% of all the AGEs [52]. Figure 3B indicates that the amount of dicarbonyl compounds formed raised with increasing incubation time. The formation of dicarbonyl compounds was significantly attenuated by *S. orientalis* extracts. The inhibitory activity was in the order of SE-EA > SE-Hex > SE > SE-MeOH. On day 7, the suppression rate of these extracts were 61.9%, 47.3%, 46.5% and 28.2%, respectively.

The above data shows that *S. orientalis* extracts has slight inhibitory effects on Amadori products formation (from the NBT reduction analysis), but high inhibitory activity on dicarbonyl compound production (from the Girard-T assay). The non-enzymatic glycation reaction of protein for the formation of AGEs can be classified into three stages [52]. In the first stage, reducing sugars condense with the free amino groups of proteins to form Schiff bases, and then produce Amadori products. In the second stage, the Amadori products further react with dicarbonyl compounds such as glyoxal, glycolaldehyde and methylglyoxal. In the third stage, the dicarbonyl compounds react with amino groups to form AGEs. The experimental results of the present study illustrate that *S. orientalis* extracts can retard the glycation reaction, and its degree of inhibition of the latter stages was higher than the first stage.

Several studies reported that a good correlation exists between TPC, TFC, antioxidant activity and the inhibition of AGEs formation for some plants [16,44,52]. For *S. orientalis* extracts, only the correlations between TPC and reducing power with the suppression on dicarbonyl compounds production were high (R = 0.804 and 0.914, respectively), and others were insignificant (Table 1). Among the four extracts, SE-EA had high TPC, TFC, antioxidant activity, as well as high ability to inhibit the formation of glycation end products.

### 2.4. Inhibitory Effects on Carbohydrate-Hydrolyzing Enzymes

Type 2 diabetes is often caused by the inappropriate regulation of carbohydrate and lipid metabolism, leading to elevated postprandial blood sugar. The digestion of starch by pancreatic α-amylase and uptake of glucose by intestinal α-glucosidase would cause a sudden rise in blood glucose level, resulting in hyperglycemia in Type 2 diabetes patients. An effective strategy for Type 2 diabetes management is the inhibition on intestinal α-glucosidase and pancreatic α-amylase [53,54].

**Figure 4:**
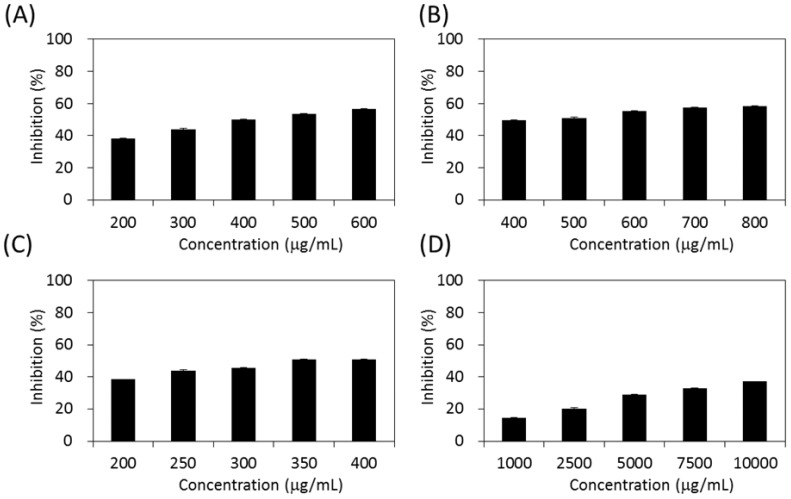
Inhibitory activities of *S. orientalis* extracts on α-glucosidase. (**A**) SE; (**B**) SE-Hex; (**C**) SE-EA; and (**D**) SE-MeOH.

As shown in Figure 4, the four extracts of *S. orientalis* exhibited inhibitory effects on α-glucosidase in a dose-dependent manner. The inhibition activity followed the order of SE-EA (IC_50_ = 362.3 ± 9.2 μg/mL) > SE-Hex (IC_50_ = 424.6 ± 11.6 μg/mL) > SE (IC_50_ = 429.3 ± 12.1 μg/mL), whereas the inhibitory activity of SE-MeOH was low (IC_50_ > 10,000 μg/mL). By comparison, the IC_50_ value of the positive control, acarbose, was 27.7 ± 0.5 μg/mL.

**Figure 5:**
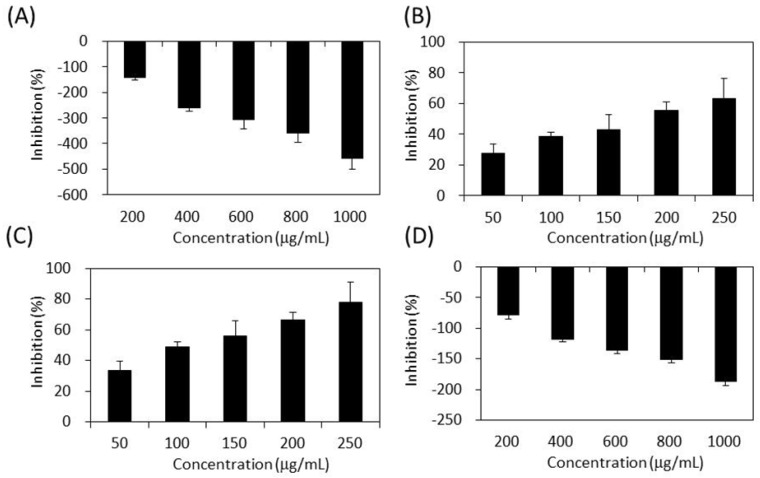
Inhibitory activities of *S. orientalis* extracts on α-amylase. (**A**) SE; (**B**) SE-Hex; (**C**) SE-EA; and (**D**) SE-MeOH.

Figure 5 demonstrates the effects of *S. orientalis* extracts on α-amylase. Only SE-EA (IC_50_ = 119.0 ± 17.7 μg/mL) and SE-Hex (IC_50_ = 175.8 ± 18.6 g/mL) exhibited the suppressive effects, whereas the IC_50_ value of acarbose was 25.4 ± 0.6 μg/mL. The SE and SE-MeOH enhanced the activity of α-amylase (the inhibition values are negative in Figure 5). This opposite result may result from the difference in the chemical ingredients contained in each extract, rather than the difference in TPC or TFC.

Several studies have shown the good correlation between TPC and antiglycemic activity, as well as α-glucosidase and α-amylase [46,55,56]. From Table 1, the inhibitory ability of *S. orientalis* extracts on α-glucosidase correlated well with TPC, DPPH and ABTS scavenging activity, reducing power, and the suppression on dicarbonyl compounds production (R > 0.9). Due to the fact that the α-amylase inhibitory activity data was insufficient to calculate the correlations, we can only conclude that SE-EA had highest TPC, TFC, antioxidant activity and anti-AGEs formation activity, as well as possessed the most potent α-amylase and α-glucosidase inhibitory activity.

### 2.5. Inhibitory Effect on Lipase

**Figure 6:**
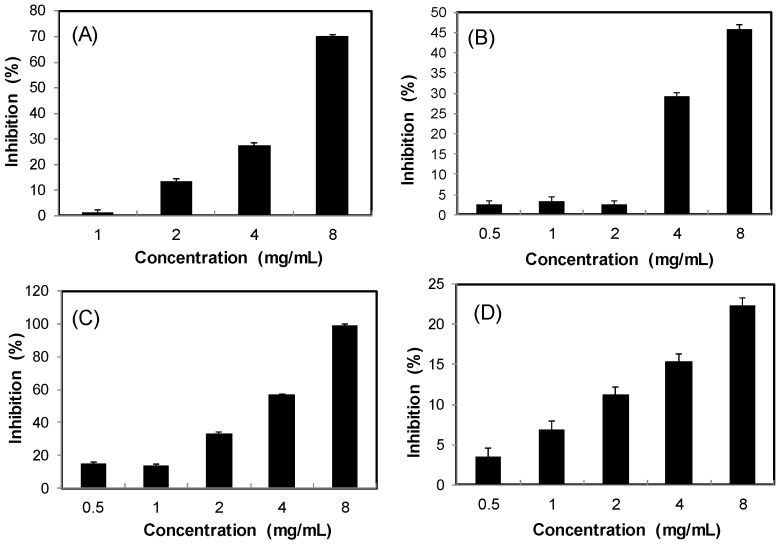
Inhibitory activities of *S. orientalis* extracts on pancreatic lipase. (**A**) SE; (**B**) SE-Hex; (**C**) SE-EA; and (**D**) SE-MeOH.

Obesity is a key factor contributing to the metabolic syndrome. Inhibition of dietary lipid absorption through the suppression of pancreatic lipase activity is an effective approach to the management of obesity and hyperlipidaemia [18]. Figure 6 shows that the four *S. orientalis* extracts inhibited lipase activity dose-dependently. SE-EA extract exhibited the highest suppression activity on lipase (IC_50_ = 3.67 ± 0.52 mg/mL), followed by SE (IC_50_ = 6.04 ± 0.68 mg/mL). On the other hand, SE-Hex and SE-MeOH showed weak effects on lipase. By comparison, the IC_50_ value of the positive control, orlistat, was 0.064 ± 0.005 μg/mL.

Several studies have reported that natural polyphenols could inhibit pancreatic lipase [18,57,58]. Table 1 demonstrates that the inhibitory effects on lipase of *S. orientalis* extracts correlated well with TPC, TFC, DPPH scavenging activity, reducing power, suppression of dicarbonyl compounds formation, and the inhibition activity on α-glucosidase (R = 0.813–0.985). However, it is worth noting that the *S. orientalis* extracts had much higher inhibitory activity (lower IC_50_ values) toward α-glucosidase and α-amylase than lipase. These results suggest that *S. orientalis* extracts can be an excellent source as antiglycemic inhibitors to manage postprandial blood glucose level, but have weak effects on the control of body weight and obesity. 

### 2.6. Inhibitory Effect on ACE

ACE inhibitors have been widely developed to prevent angiotensin II production in cardiovascular diseases and to treat hypertension related to metabolic syndrome. Several herbal medicines and food plants have been claimed to be effective in inhibiting ACE [59,60,61,62].

**Figure 7:**
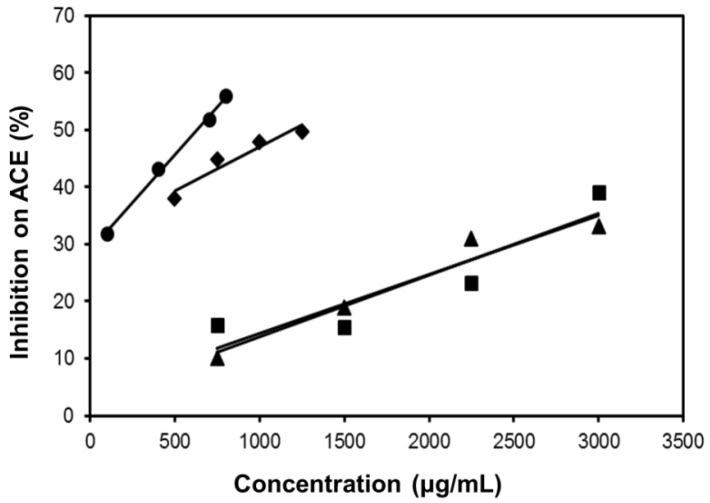
Inhibitory activities of *S. orientalis* extracts on ACE. (●) SE-EA; (♦) SE; (■) SE-Hex; and (▲) SE-MeOH.

**Figure 8:**
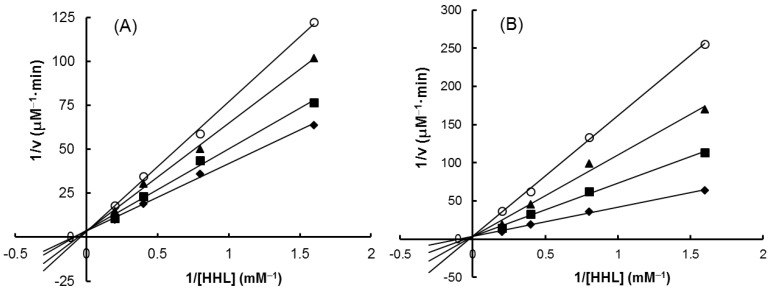
Lineweaver–Burk plots on ACE-inhibitory activity. (**A**) SE-EA: (♦) 0 μg/mL; (■) 500 μg/mL; (▲) 600 μg/mL; (O) 700 μg/mL. (**B**) Captopril: (♦) 0 μg/mL; (■) 0.001 μg/mL; (▲) 0.002 μg/mL; (O) 0.003 μg/mL.

As shown in Figure 7, all the four extracts of *S. orientalis* inhibited ACE activity dose-dependently. SE-EA showed the most significant effect (IC_50_ = 626.6 ± 15.0 μg/mL), followed by SE (IC_50_ = 1197.9 ± 26.4 μg/mL), whereas the other two extracts had only slight suppression activities. By comparison, the IC_50_ value of the positive control, captopril, was 2.69 ± 0.11 ng/mL. The inhibition type of SE-EA and captopril on ACE was examined by the Lineweaver-Burk plot technique (Figure 8). Table 2 summarized the kinetic type and the values of the related constants. Both of the SE-EA and captopril showed competitive inhibition type, i.e., the test samples competing with the substrate for binding to the active site of the enzyme. The K_i_ value of captopril (1.0 × 10^−3^ μg/mL) was much smaller than that of SE-EA (794.5 μg/mL) showing the drug has much stronger affinity to the ACE active site than SE-EA.

From Table 1, except for the poor correlation with the inhibitory capabilities on Amadori products formation and α-glucosidase, the ACE suppression activity of *S. orientalis* extracts correlated well with all other parameters (R = 0.823–0.983).

### 2.7. Chemical Composition of S. orientalis Extracts

**Figure 9:**
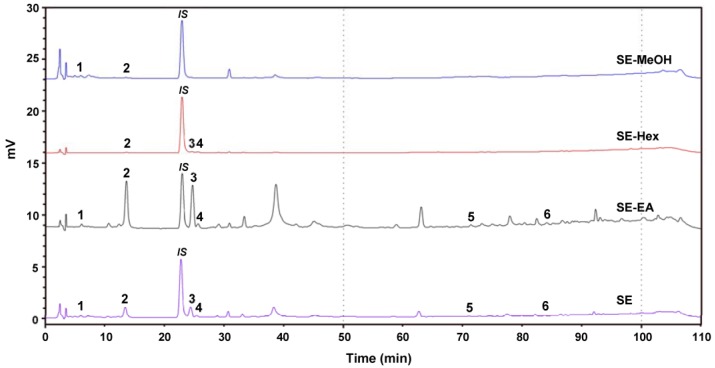
High performance liquid chromatography (HPLC) chromatogram of the four extracts of *S. orientalis*. Identified components: 1, chlorogenic acid; 2, syringic acid; 3, *p*-coumaric acid; 4, syringaldehyde; 5, luteolin; 6, apigenin. The peak denoted as “IS” is the internal standard.

The phenolic compounds of the *S. orientalis* extracts were analyzed by HPLC (Figure 9). Six components were identified as chlorogenic acid (peak 1, retention time (RT) = 7.7 min), syringic acid (peak 2, RT = 14.5 min), *p*-coumaric acid (peak 3, RT = 26.2 min), syringaldehyde (peak 4, RT = 26.7 min), luteolin (peak 5, RT = 71.7 min), and apigenin (peak 6, RT = 84.0 min). Some plant extracts containing these same phenolic compounds were reported to have the potent antioxidant, antihyperglycemic, antihyperlipidemia and/or antihypertensive activities [63,64,65,66,67,68]. Table 3 shows that the SE-EA contained the highest amounts of syringic acid, *p*-coumaric acid and syringaldehyde among these four extracts, which partly explains why the SE-EA extract had the best bioactivities.

## 3. Materials and Methods

### 3.1. Chemicals and Reagents

The *S. orientalis* linne plant materials were bought from Yuanshan Company (Kaohsiung City, Taiwan), and its nucleotide sequence was determined and deposited in the GenBank database with accession number JN987228 [69]. Acarbose, α-amylase (porcine pancreatic Type IV-B), angiotensin converting enzyme (ACE, rabbit lung), l-ascorbic acid, 2,2′-azino-bis(3-ethylbenzothiazoline-6-sulphonic acid) (ABTS), 2,6-bis(1,1-dimethylethyl)-4-methylphenol (BHT), captopril, (+)-catechin, 3,5-dinitrosalicylic acid (DNS), 1,1-diphenyl-2-picrylhydrazyl (DPPH), DL-dithiothreitol (DTT), Folin-Ciocalteu reagent, gallic acid, Girard’s reagent T, glucose, α-glucosidase (*Saccharomyces cerevisiae*), glyoxal, Hippuryl-His-Leu acetate salt (HHL), lipase (porcine pancrease Type II), nitroblue tetrazolium (NBT), 4-nitrophenyl-α-d-glucopyranoside (PNPG), *p*-nitrophenyl laurate, orlistat, and trichloroacetic acid (TCA) were purchased from Sigma-Aldrich Chemicals (St. Louis, MO, USA). Bovine serum albumin (BSA) was purchased from Fluka Biochemika (Buchs, Switzerland). All other chemicals were of reagent or analytical grade. 

### 3.2. Preparation of S. orientalis Extracts

Dry aerial parts of *S. orientalis* (9.3 kg) were crushed and soaked in 95% ethanol (47 L) for one day, and then extracted another two times with 47 L ethanol each. The extracted solutions were collected and filtered. The solvent was removed with a vacuum evaporator. The residue was then dried in a freeze-dryer. Total dry mass of this crude extract (SE) was 489 g, and the yield was 5.3%. The SE extract (50 g) was further extracted sequentially with *n*-hexane (SE-Hex), ethyl acetate (SE-EA) and methanol (SE-MeOH). The dry weights of SE-Hex, SE-EA and SE-MeOH were 19.2, 10.4 and 18.6 g, respectively. Accordingly, the yields of these three extracts from the SE extract were 38.4%, 20.8% and 37.2%, respectively.

### 3.3. TPC and TFC Analysis [51]

To analyze TPC of each extract, an aliquot of the sample solution (150 μL) was mixed with 0.2 N Folin-Ciocalteu reagent (750 μL) and 7.5% sodium carbonate solution (600 μL). The mixture was held at room temperature for 30 min and the absorbance was read at 765 nm with a spectrophotometer (Ultrospec 2100 pro, GE Healthcare, Amersham, UK). A calibration curve was obtained using gallic acid as a standard. The TPC was expressed as milligrams of gallic acid equivalents per gram of dry extract.

To analyze TFC, an aliquot of sample solution (150 μL) was mixed with distilled water (600 μL), and 15% Na_2_CO_3_ (37.5 μL). After standing at room temperature for 5 min, 10% AlCl_3_ (75 μL) was added. Six minutes later, 1 N NaOH (250 μL) and distilled water (137.5 μL) were added. The absorbance of the solution was measured against a blank at 510 nm. A calibration curve was obtained using catechin as a standard. The TFC was expressed as milligrams of catechin equivalents per gram of dry extract.

### 3.4. Antioxidant Activity Assay

#### 3.4.1. Scavenging Activity on DPPH Radicals [51] 

An ethanolic solution of DPPH (250 μL, 0.5 M) was mixed with sample solution (1.0 mL). After standing the solution in the dark for 30 min, the absorbance of the solution was measured against a control and a blank at 517 nm. The control was the measurement using ethanol to replace the sample solution in the reaction mixture. The blank was measured using ethanol to replace DPPH in the reaction mixture. The scavenging activity of the DPPH radicals with extract was calculated by the following equation:

DPPH• scavenging activity (%) = [1 − (A_sample_ − A_blank_)/A_control_] × 100% (1)

After conducting the measurements under different concentrations of sample solution, the IC_50_ value, i.e., the concentration of sample required to cause 50% inhibition, was estimated from the plot of scavenging activity against the sample concentration. The IC_50_ values were expressed as means ± sd (standard deviation) of the triplicate measurements.

#### 3.4.2. Scavenging Activity on ABTS Radicals [70]

ABTS solution (7.4 mM) and potassium persulfate solution (2.6 mM) were mixed at a 1:1 volume ratio and kept for 12–16 h in darkness at room temperature. To this mixture solution (180 μL), different concentrations of sample solution or the reference standard BHT solution (20 μL) were added and incubated for 2 h at 37 °C in the dark. The absorbance of the reaction mixture was measured at 735 nm. The control was the measurement using ethanol to replace the sample solution in the reaction mixture:

ABTS•^+^ scavenging activity (%) = (A_control_ − A_sample_)/A_control_ × 100% (2)

The IC_50_ value was estimated from the plot of scavenging activity against the sample concentration. The IC_50_ values were expressed as means ± sd of the triplicate measurements.

#### 3.4.3. Reducing Power Assay [71]

The reducing power was determined by the potassium ferricyanide-ferric chloride method. Briefly sample solution (0.1 mL) was mixed with sodium phosphate buffer (0.5 mL, 0.2 M, pH 6.6) and potassium ferricyanide (0.5 mL, 1%, *w/v*), and incubated at 50 °C for 20 min. After cooling, TCA solution (0.5 mL, 10%, *w/v*) was added and centrifuged (3000 rpm, 10 min). To supernatant (0.5 mL), distilled water (0.5 mL) and ferric chloride (0.2 mL, 0.1%, *w/v*) were added. The absorbance at 700 nm was recorded. Increased absorbance of the reaction solution indicated increased reducing power. 

### 3.5. Inhibitory Activity on AGEs Formation [47,52]

BSA (800 μL, 10 mg/mL in 50 mM phosphate buffer, pH 7.4) was mixed with 1 M glucose (160 μL) and different concentrations of sample solution (40 μL). The mixture was incubated at 80 °C for 0, 1, 3, 5, and 7 days to obtain glycated material solution. The formation of Amadori products and dicarbonyl compounds were analyzed by NBT reduction and Girard-T assay, respectively.

#### 3.5.1. Nitroblue Tetrazolium (NBT) Reductive Assay 

The glycated material solution (0.5 mL) and 0.3 mM NBT reagent (2.0 mL, in 0.1 M, pH 10.35 sodium carbonate buffer) were incubated at room temperature for 15 min, and the absorbance was measured at 530 nm against a blank.

#### 3.5.2. Girard-T Assay 

The glycated material solution (0.4 mL) was incubated with 0.5 M Girard’s reagent T solution (0.2 mL) and sodium formate (3.4 mL, 0.5 M, pH 2.9) at room temperature for 1 h. Absorbance was monitored at 295 nm against a blank. A calibration curve was prepared using glyoxal as a standard.

### 3.6. Antiglycemic Assays [44]

#### 3.6.1. Assay of α-Glucosidase Activity

α-Glucosidase from *S. cerevisiae* was dissolved in 0.1 M phosphate buffer (pH 6.9). An aliquot of sample solution (100 μL), α-glucosidase solution (20 μL, 0.4 U/mL), DTT (20 μL, 1 mM) and PNPG (20 μL, 5 mM) were mixed and incubated at 37 °C for 15 min. The reaction was stopped with 0.2 M sodium carbonate (80 μL). The absorbance was determined at 400 nm using an ELISA reader (Model 550, Bio-Rad Laboratories, Hercules, CA, USA). The percentage inhibition was calculated as follows:

% Inhibition = [(A_Control_ − A_Sample_ + A_Sample_·_Blank_)/(A_Control_ − A_Blank_)] × 100% (3)

where A_Control_ is the absorbance from the test using phosphate buffer to replace the sample solution; A_Sample_ is the absorbance from the sample solution; A_Sample Blank_ is the absorbance from the test that sodium carbonate was added before the reaction and the same steps were performed; and A_Blank_ is the absorbance from the test using phosphate buffer to replace the sample solution, sodium carbonate was added before the reaction and the same steps were conducted. The IC_50_ value was estimated from the plot of percentage inhibition against the sample concentration. The IC_50_ values were expressed as means ± sd of the triplicate measurements.

#### 3.6.2. Assay of α-Amylase Activity

Porcine pancreatic α-amylase was dissolved in distilled water to give a concentration of 2 U/mL. Potato soluble starch solution (1%) was prepared in 20 mM phosphate buffer (pH 6.9). DNS solution was prepared with DNS (1 g), potassium sodium tartrate tetrahydrate (30 g) and 2 M NaOH in 100 mL of solution. In the assay, sample solution (80 μL) and α-amylase solution (40 μL) were added and incubated at room temperature for 10 min. Then starch solution (40 μL) was added and incubated at 37 °C for 10 min. Finally, DNS solution (80 μL) was added and the mixture was incubated at 95 °C for 10 min. The absorbance was monitored at 540 nm by ELISA reader. The percentage inhibition (as Equation (3)) and the IC_50_ value were determined as described above.

### 3.7. Assay of Lipase Activity [72]

Porcine pancreas lipase was dissolved in Tris buffer (0.1 M, pH 7.5) at 10 mg/mL; then the supernatant was used after centrifugation at 4 °C, 3600 rpm for 5 min. The substrate solution was 1.6 mM *p*-nitrophenyl laurate containing 1% Triton X-100. In the assay, sample solution (50 μL) and lipase solution were mixed (50 μL), then substrate solution (100 μL) was added to start the reaction. The reaction was conducted at 37 °C for 30 min, and was heated at 85 °C for 5 min to terminate the reaction. After cooling to room temperature, the reaction mixture was centrifuged at 6000 rpm for 5 min. The absorbance of the supernantant was determined at 405 nm. The percentage inhibition (as Equation (3)) and the IC_50_ value were determined as described above.

### 3.8. Assay of ACE Activity [73]

Sample solution (75 μL) was mixed with ACE solution (75 μL, 0.1 U/mL) and kept at 37 °C for 10 min. Then, 15 mM HHL substrate solution (100 μL in 50 mM sodium borate buffer containing 0.3 M NaCl at pH 8.3) were added and the solution was reacted at 37 °C for 30 min. The reaction was stopped by adding 1 N HCl (250 μL). The product, hippuric acid, was extracted with ethyl acetate (0.75 mL). After centrifugation at 3600 rpm for 5 min, supernantant (0.5 mL) was taken and the solvent was evaporated at 80 °C for 30 min. The residue was dissolved in distilled water (1 mL) and its absorbance was measured at 228 nm. The percentage inhibition (as Equation (3)) and IC_50_ value were determined as described above. Since the extinction coefficient (K) of hippuric acid is 9.8 [73], the reaction rate of ACE (*v*) can be calculated by the following equation:

(4) $$v=\frac{A\times K}{t}$$

where A is the absorbance measured after the reaction, and t is the reaction time. The enzymatic kinetic constants V_m_, K_m_ and K_i_ values were estimated by Lineweaver-Bruk double reciprocal plot of 1/*v* vs. 1/[HHL].

### 3.9. Analysis of Chemical Compositions by HPLC

The amounts of flavonoids were determined by a Shimadzu HPLC system (Shimadzu, Kyoto, Japan) equipped with a UV detector and a C18 column (5 μm, 250 mm × 4.6 mm; Supelco, Bellefonte, PA, USA), using a gradient of acetonitrile (solvent A) and 0.1% acetic acid in water (solvent B) as mobile solvents. The extracts were dissolved in methanol and filtered with a 0.22 μm filter. The solvent gradient consisted of 0–100 min (12–40% A) and 100–110 min (40–12% A). The flow rate was 1.0 mL/min, and the sample injection size was 20 μL. The detection was carried out at 345 nm. The compounds were identified by comparison of their retention time with those of reference compounds. Quantification was performed by the construction of standard curves obtained by linear regression using the Microsoft Excel software (Microsoft Inc., Redmond, WA, USA).

### 3.10. Statistical Analysis

All experiments were conducted for three to five independent replicates. The data are expressed in terms of means ± sd. Correlation between any two parameters was determined by the linearity of these two parameters and was expressed by the correlation coefficients (R). The experimental data were analyzed using Microsoft Excel. 

## 4. Conclusions

This study provides insights into the potential of *S. orientalis* extracts to inhibit key enzymes relevant to metabolic syndrome, such as Type 2 diabetes-associated hyperglycemia, obesity and hypertension in connection with their phenolic contents, antioxidant activities and AGEs formation. Our results revealed that SE-EA extract exhibited good inhibitory activities on α-amylase, α-glucosidase, lipase and ACE, as well as highest DPPH, ABTS scavenging activities, reducing power and prevented the AGEs formation. This implies that SE-EA extract has the potential to be a therapeutic agent for the prevention or treatment of metabolic syndrome. Additionally, we observed that high correlations existed among the parameters of TPC, antioxidant activities, inhibitory activities on α-amylase, α-glucosidase, lipase and ACE (R > 0.9). Moderate correlations were found among the parameters of TFC, antioxidant activities, and suppression of dicarbonyl compounds formation (R = 0.5–0.9). While only slight or inversely relationships existed between the inhibitory effect on Amadori products formation and other activities.

To our knowledge, this is the first report on an evaluation of the feasibility of using *S. orientalis* extracts in the prevention or management of metabolic syndrome. Based on the above results, this study discloses a biochemical rationale for further animal and clinical studies on *S. orientalis* extracts. Further work is still needed to fully elucidate the details of *in vivo* inhibitory activities, the related mechanisms involved, and to examine its bioactive constituents.

## Figures and Tables

**Table 1:** Correlations between antioxidant, anti-AGEs formation, anti-hyperglycemic, antihyperlipidemia and antihypertension activities in *S. orientalis* extracts with their respective total polyphenols (TPC) and total flavonoids (TFC) contents.

Parameter	Antioxidation			Anti-AGEs Formation		Antihyperglycemic		Antihyper-Lipidemia	Antihyper-Tension
	1/IC_50, DPPH_	1/IC_50, ABTS_	Reducing Power	NBT Reduction	Girard-T Assay	1/IC_50, amy_	1/IC_50, glu_	1/IC_50, PL_	1/IC_50, ACE_
TPC	0.993	0.925	0.975	−0.346	0.804	-	0.945	0.945	0.983
TFC	0.907	0.829	0.794	0.042	0.585	-	0.650	0.813	0.926
1/IC_50, DPPH_	1	0.953	0.944	−0.337	0.735	-	0.919	0.910	0.978
1/IC_50, ABTS_		1	0.828	−0.522	0.530	-	0.949	0.750	0.870
Reducing power			1	−0.311	0.914	-	0.977	0.985	0.965
NBT reduction				1	−0.121	-	−0.797	−0.146	−0.170
Girard-T assay					1	-	1.000	0.943	0.823
1/IC_50, amy_						1	-	-	-
1/IC_50, glu_							1	0.847	0.686
1/IC_50, PL_								1	0.965
1/IC_50, ACE_									1

**Table 2:** The inhibitory properties of SE-EA on ACE in reference to captopril.

Sample	IC_50_ (μg/mL)	K_i_ * (μg/mL)	Inhibition Type
SE-EA	626.6	794.5	Competitive
Captopril	2.69 × 10^−3^	1.0 × 10^−3^	Competitive

*Inhibition constant K_i_ were calculated from Lineweaver–Burk plots.

**Table 3:** Phenolic compounds of the four extracts of *S. orientalis*.

Peak No.	Compound	Concentration (mg/g Extract)			
		SE-MeOH	SE-Hex	SE-EA	SE
1	Chlorogenic acid	0.95 ± 0.05	- ^b^	0.72 ± 0.04	0.98 ± 0.04
2	Syringic acid	TA ^a^	TA ^a^	2.21 ± 0.11	0.26 ± 0.02
3	*p*-Coumaric acid	- ^b^	TA ^a^	1.76 ± 0.08	0.71 ± 0.03
4	Syringaldehyde	- ^b^	0.34 ± 0.02	0.57 ± 0.03	0.39 ± 0.02
5	Luteolin	- ^b^	- ^b^	TA ^a^	TA ^a^
6	Apigenin	- ^b^	- ^b^	TA ^a^	TA ^a^

^a^ TA: Trace amount; ^b^ -: Not detectable.

## Abbreviations

The following abbreviations are used in this manuscript:

ABTS	2,2′-Azino-bis(3-ethylbenzothiazoline-6-sulphonic acid)
ACE	Angiotensin converting enzyme
AGEs	Advanced glycated end products
BHT	2,6-Bis(1,1-dimethylethyl)-4-methylphenol
BSA	Bovine serum albumin
DNS	3,5-Dinitrosalicylic acid
DPPH	1,1-Diphenyl-2-picrylhydrazyl
DTT	d,l-Dithiothreitol
HHL	Hippuryl-His-Leu acetate salt
NBT	Nitroblue tetrazolium
PNPG	4-Nitrophenyl-α-d-glucopyranoside
ROS	Reactive oxygen species
RT	Retention time
SE	Ethanol extract of *S. orientalis*
SE-EA	Ethyl acetate extract of *S. orientalis*
SE-Hex	*n*-Hexane extract of *S. orientalis*
SE-MeOH	methanol extract of *S. orientalis*
TCA	Trichloroacetic acid
TFC	Total flavonoids content
TPC	Total polyphenols content
